# Urogenital schistosomiasis in three different water access in the Senegal river basin: prevalence and monitoring praziquantel efficacy and re-infection levels

**DOI:** 10.1186/s12879-022-07813-5

**Published:** 2022-12-29

**Authors:** Bruno Senghor, Eglantine Mathieu-Begné, Olivier Rey, Souleymane Doucouré, Doudou Sow, Bocar Diop, Mariama Sène, Jérôme Boissier, Cheikh Sokhna

**Affiliations:** 1 Campus International IRD-UCAD de Hann, Vectors-Tropical and Mediterranean Infections (VITROME) Laboratory, 1386 Dakar, Senegal; 2 Host Pathogen Environments Interactions (IHPE) Laboratory, CNRS, IFREMER, University of Montpellier, University of Perpignan via Domitia, Perpignan, France; 3 Department of Parasitology-Mycology, UFR of Health Sciences, University Gaston Berger, 234, Saint-Louis, Senegal; 4 Laboratory of Biological and Agronomic Sciences and Modelling of Complex Systems, UFRS2ATA, Gaston Berger University of Saint-Louis, Saint-Louis, Senegal; 5 National Schistosomiasis Control Program (NSCP), Ministry of Health, Dakar, Senegal; 6 VITROME, IRD, SSA, AP-HM, IHU-Mediterranean Infection, Aix-Marseille Univ, Marseille, France

**Keywords:** Urinary schistosomiasis, *S. haematobium*, Praziquantel, Drug efficacy, Re-infection, School children, Senegal river basin, Senegal

## Abstract

**Background:**

Urogenital schistosomiasis is a neglected tropical disease most prevalent in sub-Saharan Africa. In the Senegal river basin, the construction of the Diama dam led to an increase and endemicity of schistosomiasis. Since 2009, praziquantel has frequently been used as preventive chemotherapy in the form of mass administration to Senegalese school-aged children without monitoring of the treatment efficacy and the prevalence after re-infection. This study aims to determine the current prevalence of urogenital schistosomiasis (caused by *Schistosoma haematobium*), the efficacy of praziquantel, and the re-infection rates in children from five villages with different water access.

**Methods:**

The baseline prevalence of *S. haematobium* was determined in August 2020 in 777 children between 5 and 11 years old and a single dose of praziquantel (40 mg/kg) was administered to those positive. The efficacy of praziquantel and the re-infection rates were monitored 4 weeks and 7 months after treatment, respectively, in 226 children with a high intensity of infection at baseline.

**Results:**

At the baseline, prevalence was low among children from the village of Mbane who live close to the Lac de Guiers (38%), moderate among those from the villages of Dioundou and Khodit, which neighbor the Doue river (46%), and very high at Khodit (90.6%) and Guia (91.2%) which mainly use an irrigation canal. After treatment, the observed cure rates confirmed the efficacy of praziquantel. The lowest cure rate (88.5%) was obtained in the village using the irrigation canal, while high cure rates were obtained in those using the lake (96.5%) and the river (98%). However, high egg reduction rates (between 96.7 and 99.7%) were obtained in all the villages. The re-infection was significantly higher in the village using the canal (42.5%) than in the villages accessing the Lac de Guiers (18.3%) and the Doue river (14.8%).

**Conclusion:**

Praziquantel has an impact on reducing the prevalence and intensity of urogenital schistosomiasis. However, in the Senegal river basin, *S. haematobium* remains a real health problem for children living in the villages near the irrigation canals, despite regular treatment, while prevalence is declining from those frequenting the river and the Lac de Guiers.

*Trial registration* ClinicalTrials.gov, NCT04635553*.* Registered 19 November 2020 retrospectively registered, https://www.clinicaltrials.gov/ct2/show/NCT04635553?cntry=SN&draw=2&rank=4

**Supplementary Information:**

The online version contains supplementary material available at 10.1186/s12879-022-07813-5.

## Background

Human schistosomiasis is a neglected tropical disease which affects more than 230 million people worldwide [1] and accounts for an estimated 1.9 million disability-adjusted life years annually [2]. The disease is mostly prevalent in sub-Sharan Africa (SSA), where up to 90% of the world’s total cases live, leading to an estimate of 280,000 deaths each year [3]. Human schistosomiasis is caused by five species of flatworm parasites belonging to the *Schistosoma* genus. The two most notable species, *Schistosoma haematobium* and *Schistosoma mansoni* are predominant in SSA and cause urogenital and intestinal schistosomiasis, respectively [4]. People get infected during occupational, domestic or recreational activities in contact with freshwater containing infested snail intermediate hosts [5].

*Schistosoma* infections can be treated by the praziquantel (PZQ), a drug routinely used at the standard oral dose of 40 mg/kg body weight [6]. In the past 10 years, PZQ has been repeatedly administered in sub-Saharan Africa through National Schistosomiasis Control Programs (NSCP), to children aged between 5 and 14, to prevent them from developing severe, late-stage chronic forms of the disease [7, 8]. Between 2010 and 2019, the total number of people treated has increased considerably from 33 to 105.4 million and the number of tablets for the 2020 campaigns was estimated at 226 million [9]. Although globally efficient, this ongoing and repeated mass drug administration (MDA) acts as an important drug pressure which could lead to the development of schistosome strains which are resistant to the PZQ [10]. Several studies have monitored the efficacy of PZQ in field populations in endemic countries. The majority of these studies indicated a considerable reduction of morbidity and transmission of schistosomiasis, with a high cure rates and satisfactory eggs reduction rates [11, 12], while some reported treatment failure based on low CR and ERR [13]. None of them has confirmed the existence of schistosome resistance to the PZQ, but there is always a need to continuously monitor PZQ efficacy in various epidemiological situations.

In Senegal, prior to the Diama dam being constructed in 1986 to prevent the intrusion of seawater into the Senegal river, intestinal schistosomiasis had never been reported in the Senegal river delta [14], and only a low prevalence of *S. haematobium* (average 10.4%) had been found in villages situated in the higher land away from the river, where the transmission sites were rain pools [15]. The same epidemiological situation was described by Vercruysse et al*.* in 1985 [16], in several villages near the Lampsar river in the lower valley and in the Podor area in the middle valley, where the prevalence of haematuria varied between 0 and 33%. Once the Diama dam was operational, extensive water development took place in the Senegal river basin, resulting in a large increase in the amount of freshwater available for irrigation [17]. Two years later, Talla et al*.* reported for the first time the presence of *S. mansoni* in the town of Richard Toll [14]. More generally, such major changes to the Senegal river basin scale led to the expansion of the population of snails which are schistosomes’ intermediate hosts, which led subsequently to a major outbreak of intestinal schistosomiasis [14, 18–20] and an increase in the prevalence of urinary schistosomiasis [21]. Over the same period, the Manantali dam was completed upstream in Mali, to control the flow of the Senegal river and to generate electricity. It contributed significantly to the observed increase in the infection intensity of urinary schistosomiasis in the middle valley, where prevalence reached 55% within 7 years [22] and up to between 95.5 and 100% between 2006 and 2007 [23, 24]. Since 2009, regular MDA programmes by the Senegalese NSCP have been conducted in several endemic villages along the Senegal river basin [25], but the disease still constitutes a public health problem in certain localities where a high prevalence of urogenital schistosomiasis has been observed, including in the town of Richard Toll (87%) and around the Lac de Guiers (88%) area [26]. The efficacy of PZQ against *S. haematobium* and *S. mansoni* and the patterns of re-infection in this region have been evaluated by previous studies [23, 27–30]. Historically, PZQ resulted in generally low cure rates (18–42.5%) and a moderate reduction of infection intensity, especially for *S. mansoni* [28–30]. In more recent studies, a high reduction of infection intensity (between 97–98%) was achieved, with high cure rates (81–95%) after a single dose of PZQ (40 mg/kg) for *S. mansoni* and a 100% cure rate after a second dose at a 6 week interval for *S. haematobium* [23, 27]. This variability in response to schistosome parasites to the drug sparked controversy over possible resistance to PZQ, especially for *S. mansoni* in Senegal [13]. The high egg loads before treatment, as well as the rapid re-infection rate, were evoked to explain the poor response of the parasites to PZQ [4, 20, 23]. However, the possibility of emerging PZQ-resistant schistosome strains remains a global concern [31–33] and the efficacy of treatment in the field needs to be continuously monitored in areas undertaking MDA. In the Senegal river basin, schistosomiasis transmission occurs in three major epidemiological systems, depending on the type of water access: the Senegal river and its tributaries, the Lac de Guiers, and irrigation canals. The dynamics of schistosomiasis transmission can vary from one type to another, mainly due to differences in the snail intermediate hosts’ dynamics [34, 35], the extent and accessibility of the transmission sites [36], the nature of the water contact behaviour [37], and the unequal exposure of the population [38]. The aim of this study was primarily to evaluate the prevalence of *S. haematobium* among these systems and, secondarily, to monitor the efficacy of a single dose of PZQ and the pattern of re-infection in five villages located in these three epidemiological systems in the Senegal river basin.

## Methods

### Study area and villages

**Fig. 1:**
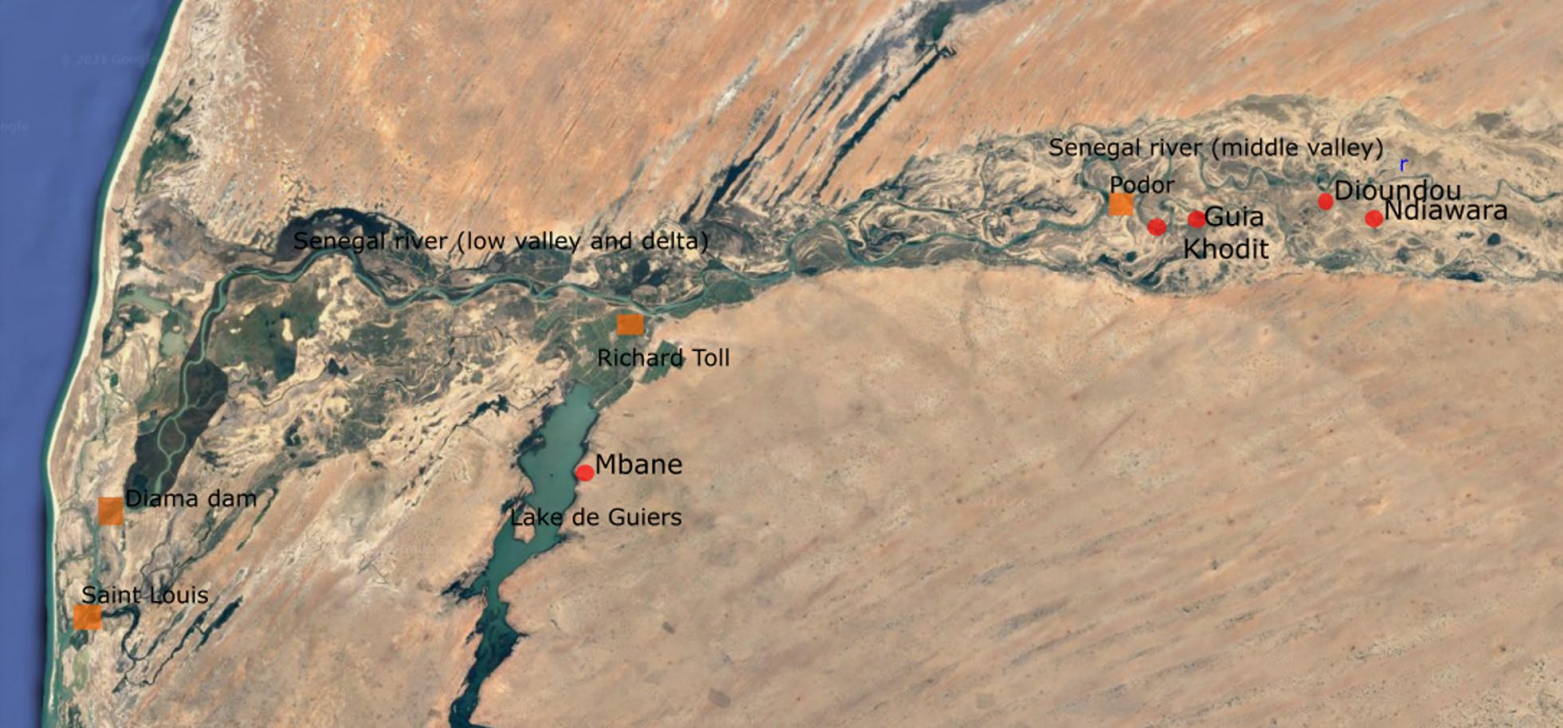
Satellite map of the study area showing the location of the study villages (red dots) in northern Senegal. The GPS coordinates of the villages are: Mbane (16° 16′ 15″ N 15° 48′ 07″ W), Ndiawara (6° 35′ 05″ N 15° 51′ 2″ W), Dioundou (6° 36′ 4″ N 14° 54′ 31″ W), Guia (16° 35′ 53″ N 14° 55′ 23.2″ W) and Khodit (16° 35′ 44.2″ N 14° 56′ 41.1″ W)

The study was carried out in the area of the Senegal river basin in five villages (Mbane, Ndiawara, Dioundou, Guia, and Khodit). The Senegal river basin has three distinct regions which are characterised by distinct environmental conditions: a mountainous upper valley which runs from the source in Guinea to Bakel; a middle valley which runs from Bakel to Dagana, including Podor, and a lower valley which runs from Dagana to the delta, where the Diama dam is located. The study villages belong to two regions of the Senegal river basin and represent three different means of accessing water. The village of Mbane (16° 16′ 15″ N 15° 48′ 07″ W) is located along the Lac of Guiers in the lower valley. The villages of Ndiawara (6° 35′ 05″ N 15° 51′ 2″ W) and Dioundou (6° 36′ 4″ N 14° 54′ 31″ W) are located along a tributary of the Senegal river (Doue river), while Guia (16° 35′ 53″ N 14° 55′ 23.2″ W) and Khodit (16° 35′ 44.2″ N 14° 56′ 41.1″ W) are located between the Doue and an irrigation canal in the middle valley (Fig. 1).

#### The village of Mbane

The village of Mbane has a population of 2650 inhabitants. Water for domestic and recreational activities is mainly supplied directly from the lake. Livestock, agriculture and fishing are the main income-generating activities. Fishing is artisanal and is focused exclusively on the lake. Market gardening is the main agricultural activity and takes place close to the lakeside. The village has two primary schools, with a total of 598 pupils. There are taps and latrines in the village and pit latrines at the schools.

#### The villages of Ndiawara and Dioundou

The villages of Ndiawara and Dioundou have their economic activity mainly based on agro-pastoralism. Market gardening is practiced along the Doue river. This river is near to the houses and is mainly frequented for laundry, dishes, swimming and recreational activities. It constitutes the main transmission site for the populations of the two villages.

Ndiawara has a population of about 3842 inhabitants with one school of 367 pupils and four pit latrines. The village also has a health centre, water tower and two wells which are accessible to all villagers. Public taps are generally not operational, although some households have running tap water.

Dioundou is a small village with about 620 inhabitants and one school with four classes and a total of 50 pupils. There are three public taps which are served by the Ndiawara water tower. There is also one well and latrines in the village, and some houses have tap water. There is no health centre in the village and the populations consult at the medical centre at Ndiawara.

#### The villages of Guia and Khodit

The villages of Guia and Khodit depend on a pumping station that drains water from the river into a large irrigation canal. The villages include several farms mainly producing food which includes winter crops (market gardening near the canals that runs through the two villages), livestock and fishing. Several access points exist along the irrigation canal where people come into contact with the water and which that constitute the major source of transmission.

Guia has a population of about 4672 inhabitants and two schools with a total intake of 640 pupils. The largest (465 pupils) is located not far from the irrigation canal. There are four pit latrines in each school. There is also a water tower and two wells. Tap water is supplied to most houses. The village has a health centre serving other villages, including Khodit.

Khodit counts about 2336 inhabitants and one school which had 216 pupils. There are four pit latrines, one well and one water tap in the school and one in the village. The tap water is supplied by the Guia water tower.

### Sample selection and study design

**Fig. 2:**
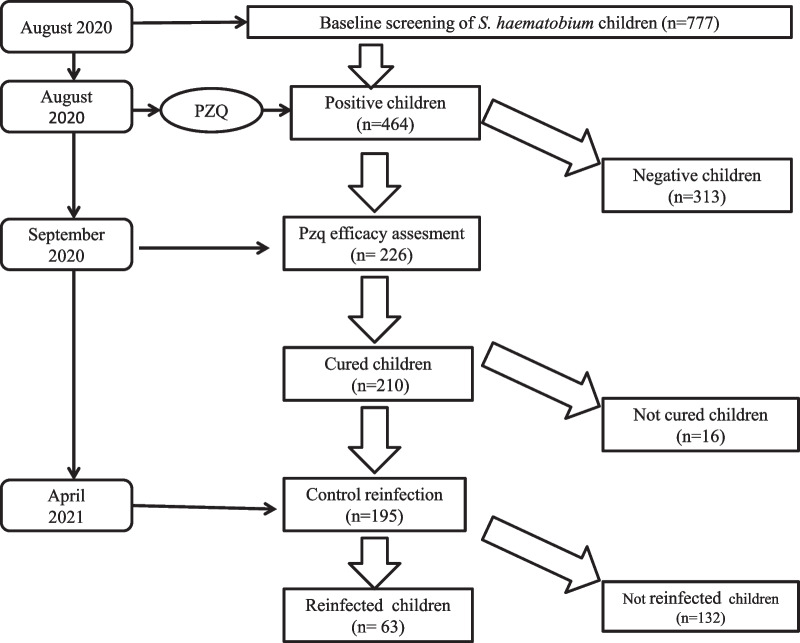
Flowchart of the study design

This study is based on a prospective cohort of children aged between 5 and 11, before and after a single treatment with PZQ (40 mg/kg) in five villages selected based on previously reported *S. haematobium* prevalence [24]. The minimum sample size for the baseline prevalence for this study was calculated based on the normal distribution N = ε2pq/i2, with N being the size of the sample; ε the normal Z score corresponding to the risk of error α = 5%; p the mean prevalence of *S. haematobium* (90%) in the villages; q = (1 − p); and i the precision, fixed at 5%. N = (1.96) × 2 × (0.9) × (0.1)/(0.05) 2 = 138. We increased this number to account for the possible loss of patients during the study that could be due to failure to sign the informed consent paperwork, the absence of children during the baseline screening, failure to provide a sample, and other reasons, by adding 5% of the minimum sample size: N = 138 + (138 × 5/100) = 145. Each village was considered as a cluster and since, at the village level, children living in the same household incur the same risks for *S. haematobium* infection, the sample size in each village (N = 145) was multiplied by two in order to limit this cluster effect. We therefore calculated the sample size for the study at 1450 children, from whom, 1054 were screened in 8 villages pre-selected, but only five villages were finally selected for the longitudinal follow-up with 777 children included in the analysis of *S. haematobium* at the baseline. As the previous prevalence was high (over 89%), we expected to have at least 50 positive children in each village (250 in total) at baseline for PZQ efficacy and re-infection monitoring, but only 226 positive children were monitored (Fig. 2).

### Sampling and data collection

The baseline sample collection took place in August 2020, 9 months after the last mass drug administration by the NSCP. Class registers were used to randomly select the study participants, taking into account the sex ratio in each school, and an identifier number was randomly allocated to each child throughout the follow-up period. Urine samples were collected in sterile 150 ml containers between 10 am and 2 pm. A urine container with the corresponding identifier was given to each child, and they were asked to go individually to the latrine and fill the container with their urine. The containers were checked to ensure that at least 10 ml could be obtained for the quantification of eggs. If the volume collected was too low, the child was asked to drink water and wait until urinating again. The container was then placed directly in a cooler in a room class to protect it from sunlight and to prevent the eggs from hatching. Once collection was completed, the cooler containing the samples were introduced in the car and transferred to the laboratory for microscopic examination on the same day.

### Baseline microscopic examination

In the laboratory, each urine sample was gently shaken to ensure homogenisation of the eggs before filtering 10 ml of the liquid through a Swinnex^®^ filter. Microscopic examination of each sample to detect *S. haematobium* eggs was performed using the filtration method [39]. The positivity of *S. haematobium* and the number of eggs per 10 ml of urine (infection intensity) were recorded. A child was considered to be infected by *S. haematobium* at baseline if at least one egg was found in their urine sample during microscopic observation. The baseline prevalence and intensity of infection of *S. haematobium* were calculated considering all participants in each village.

### PZQ treatment

The product used was Praziquantel, an existing product manufactured by Merck, SA de CV, Mexico. The drug is also used by the NSCP in MDAs in school-aged children in Senegal. Treatment was administered at the standard dose of 40 mg/kg at the health centre or at the school in each village, in accordance with Senegalese NSCP guidelines and in the presence of community relays, persons which help the health post in the MDAs at the community level. To make sure that the drug was swallowed correctly, each child was asked to open their mouth for verification. If the drugs had not been swallowed, another glass of water was given to the child. For children who vomited the drug, a second full dose was given approximately 30 min after. Children who continued to eliminate schistosome eggs (either viable or not) 1 month after the initial treatment received a second dose of 40 mg/kg.

### PZQ efficacy assessment

PZQ efficacy was determined 4 weeks after the treatment. Urine samples were collected and microscopically analysed in search of residual eggs and infection intensity was recorded, as previously described. In all samples in which calcified or non-calcified eggs were found, a hatching test to verify the egg’s viability was performed. The hatching test consisted of filtering out the remaining urine and rinsing the filter then placing the eggs in a Petri dish containing fresh water. The Petri dish was then exposed to artificial light for 30 min to allow hatching, and then checked under a dissecting microscope to detect swimming miracidia. The assessment of PZQ efficacy was measured by determining cure rate (CR) and egg reduction rate (ERR). The CR is the percentage of children who are positive at baseline and who become negative after treatment (i.e. no miracidium is found after eggs hatching in the total urine sample) and the ERR is the percentage of reduction in the arithmetic or geometric mean egg count between the baseline and after treatment [40, 41]. The ERR was calculated using the following formula: [1 − AMEC after treatment/AMEC before treatment)] × 100 [40].

### Monitoring re-infection

Re-infection was monitored 7 months after treatment, in April 2021. At this time, any child emitting parasite eggs was considered as being re-infected with *S. haematobium*. Urine samples were collected and examined as described above as eggs/10 ml urine. Re-infection was only monitored in children who were positive at baseline, received treatment and who were negative at the treatment assessment time point. Each individual was considered as being re-infected if at least one miracidium was found after the eggs had hatched.

### Data analysis

Quantitative variables (mean and standard deviation) were calculated using Microsoft Office Excel 2010. The prevalence of *S. haematobium* was calculated as the number of children who were positive over the total number screened. Intensity of infection was classified as “light” if the number of eggs/10 ml was between 1–49 eggs and “heavy” if it was more than 50 eggs [42].

At the baseline (S0), we aimed to test the effect of the type of water access, namely, irrigation canal (Guia and Khodit), lake (Mbane) or river (Dioundou and Ndiawara), and also the age and the sex of the child with regards to parasite prevalence and parasite egg load. Since our sampling design displayed an underlying spatial structure in villages, we used generalised linear mixed models (glmm) with villages set as random effect. The first model aimed at explaining the parasite prevalence (measured at the individual level, i.e. 0 or 1) and, hence, a binomial error term distribution was used. The second model aimed at explaining the parasitic load (measured at the individual level and only for children who had been parasitized), and a negative binomial error term distribution was used, since over-dispersion was detected. The two glmm were adjusted using the R package *glmmADMB* [43] and the significance of the results was examined using a type II Anova. The level of re-infection between water accesses types were compared using the Chi-squared test. In the case of a *p*-value < 0.05, the difference was considered as statistically significant.

## Results

### General population description and baseline parasitology

A total of 777 children were enrolled at baseline. The sex ratio (male/female) and the average age of participants were 0.64 and 7.9 years (Standard Deviation = 1.4), respectively. Table 1 summarises the demographic characteristic of the study population.

**Table 1:** Demographic Information of the study population

Villages	Water access	Sample size at baseline	Mean age (SD) in years	Sex ratio (M/F)
Mbane	Lac de Guiers	269	8.5 (1.07)	1.2
Ndiawara	Senegal river (Doue)	172	7.9 (1.4)	4.7
Dioundou	Senegal river (Doue)	49	7.1 (1.3)	1.3
Guia	Irrigation canal	159	7.8 (1.5)	0.4
Khodit	Irrigation canal	128	7 (1.6)	0.3
Total		777	7.9 (1.4)	0.64

Of the 777 screened children, 464 (59.7%) were infected by *S. haematobium*. Very high prevalence was found at baseline in the villages of Guia (91.2%) and Khodit (90.6%), whose inhabitants frequent the irrigation canal, while moderate prevalence was noted in the village of Ndiawara (45%), and Dioundou (49%), whose inhabitants use the river, and also in the village of Mbane (38%) which is located near Lac de Guiers (Table 2). The same trend was noted for heavy infections (more than 50 eggs/10 ml urine) where higher intensity (44% and 46.7%) were recorded in villages near the irrigation canal than other villages (Table 2).

**Table 2:** Baseline prevalence and intensity of infection of *S. haematobium* (S0) according to village, sex and age

Variables	Prevalence of *S. haematobium*	95% confidence interval	Heavy infection (more than 50 eggs/10 ml)	Light infection (1–49 eggs/10 ml)
Mbane	38% (102/269)	32.1–43.7	27 (26.5%)	75 (73.5%)
Ndiawara	45% (77/172)	37.3–52.2	23 (30%)	54 (70%)
Dioundou	49% (24/49)	34.8–63.1	5 (20.8%)	19 (79.1%)
Khodit	90.6% (116/128)	85.5–95.7	51 (44%)	65 (56%)
Guia	91.2% (145/159)	86.8–95.6	68 (46.9%)	77 (53.1%)
Male	55. 2% (169/306)	49.6–61	58 (34.3%)	111 (65.7%)
Female	62. 6% (295/471)	57.9–66.9	116 (39.3%)	179 (60.7%)
5–8 years	61. 3% (277/452)	56.6–65.8	110 (39.7%)	167 (60.3%)
9–11 years	57. 5% (187/325)	52–62.9	64 (34.2%)	123 (65.8%)

Significant differences in the prevalence and intensity of infection were noted between the types of water access (Chi-square = 172.472, df = 2, *p*-value < 0.001; F = 18.90, df = 2, *p*-value < 0.001, respectively) (Additional file 1: Fig. S1 and Additional file 2: Table S1). With regards to sex, the prevalence and intensity of infection were higher in females (62.6% and 39.3% respectively) than in males (55.2% and 34.3%, respectively) (Table 2), but the difference was not significant (Chi-square = 0.9714, df = 1, *p*-value = 0.424; F = 0.0253, df = 1, *p*-value = 0.874, respectively) (Additional file 2: Table S1). When classifying the population into two age groups, the prevalence and intensity of infection were higher in children aged 5 to 8 years (61.3% and 39.7%, respectively) than those aged 9 to 11 years (57.5% and 34.2%, respectively) (Table 2), with no significant difference (Chi-square = 0.9714, df = 1, *p*-value = 0.324; F = 0.003, df = 1, *p*-value = 0.960, respectively) (Additional file 2: Table S1).

### Treatment and efficacy of PZQ

**Fig. 3:**
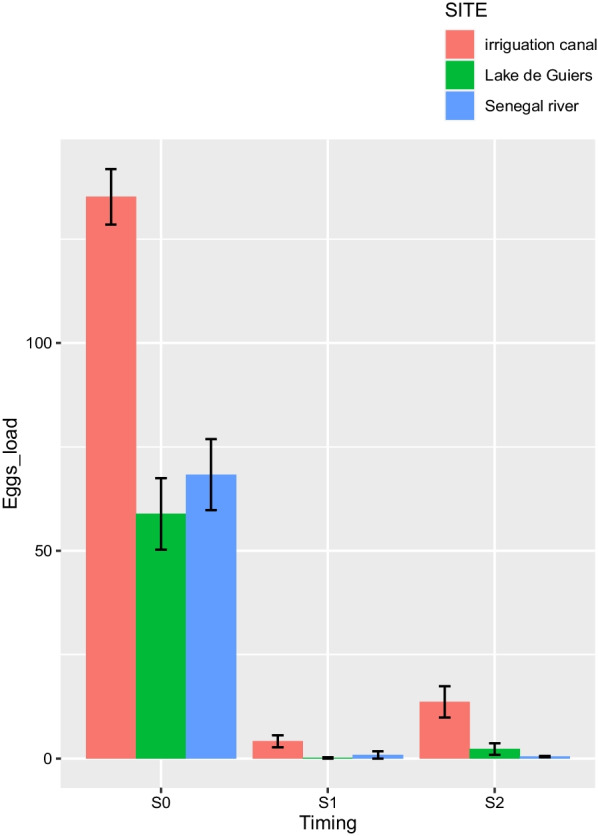
Evolution of egg loads before (S0) and 4 weeks after treatment (S1), and after 7 months (re-infection) (S2) at the different sites according to the type of water access (lake, river, irrigation canal)

All the 464 children who were infected at the first screening step received one dose of 40 mg/kg of PZQ, but only 226 with a moderate to high baseline infection and from whom informed consent was obtained were screened for PZQ efficacy (Table 3). Of the 226 children, 210 (92.9%) were negative for *S. haematobium* eggs 4 weeks after treatment, while 16 (7.1%) continued to excrete viable eggs (Fig. 2). Of the 16 children who were not completely cured, 13 live in the villages near the irrigation canal (six in Guia and seven in Khodit) and had a high infection at baseline. The other three children live in the villages near the river (two of whom had a high infection) and the Lac de Guiers (one with a light infection). The overall CR and ERR were high (92.9% and 97.16%, respectively). The lowest CR (88.8%) was obtained in children from the villages bordering the irrigation canal, with high baseline infection intensity, while the highest CRs were recorded in children from the villages along the Lac de Guiers (99.8%) and the river (98.7%). However, the ERRs were high in all the villages, with rates of 96.9%, 98.8% and 99.8% in the villages using the canal, the river and the lac de Guiers, respectively. One month after PZQ treatment, a very high decrease in the arithmetic mean was recorded in all children living in the three epidemiological systems, from 68.31 to 0.88, 58.87 to 0.14 and 135.17 to 4.16 at the Lac De Guiers, the river and the irrigation canal, respectively (Table 3). The change in the intensity of infection (mean egg load) before and after treatment is presented in Fig. 3.

**Table 3:** PZQ efficacy against *S haematobium* and re-infection according to the type of water access

*S. haematobium* infection				
Water access	Lac de Guiers (Mbane)	Senegal river (Dioundou and Ndiawara)	Irrigation canal (Guia and Khodit)	Total
PZQ efficacy (S1)				
Number of children assessed	51	58	117	226
Arithmetic mean egg count before treatment	58.87	68.31	135.17	100.02
No. of children cured	50	56	104	210
No. of children not cured	1	2	13	16
Cure rate (%)	98%	96.5%	88.8%	92.9%
Arithmetic mean egg count after treatment	0.14	0.88	4.16	2.37
Egg reduction rate (%)	99.76%	98.71%	96.92%	97.16%
Re-infection assessment (S2)				
No. of children screened	48	53	94	195
No. of children reinfected (%)	9 (18.7%)	10 (18.8%)	44 (46.8%)	63 (32.3%)
Arithmetic Mean egg count	2.28	0.46	13.62	7.56

### Re-infection

Of the 210 children who were completely negative 1 month after treatment, 195 were still present in the villages 7 months after treatment and were diagnosed for potential re-infection (i.e. the presence of viable eggs in the urine sample). Of them, 63 (32.3%) had at least one miracidium after the egg viability test was conducted and considered as being re-infected. Re-infection was recorded in all the villages regardless of water access, but children from the villages of Guia and Khodit who frequented the irrigation canal were twice as likely to be reinfected (46.8%) than children from the villages near Lac de Guiers (18.7%) and the river (18.8%). Significant differences in re-infection rates were noted between children from the villages close to the irrigation canal and those from the villages along the Lac de Guiers or the river (Chi-square = 5.2794, *p*-value = 0.021579). Conversely, no significant difference was observed in the re-infection patterns among children from villages neighbouring the lake and the river (Chi-square = 0.0002, *p*-value = 0.990012) (Table 3).

Compared to the baseline, the infection intensity remained low, and the new arithmetic mean of the eggs counts varied from 0.4% in the villages using the river to 13.62% in those frequenting the irrigation canal. The overall change in the intensity of infection (egg load) between the baseline (S1), after treatment (S1), and after re-infection (S2) is illustrated in Fig. 3.

## Discussion

The first objective of this study was to determine the current prevalence of *S. haematobium* and the intensity of infection, which are indicators of morbidity in five villages divided into three different epidemiological systems close to the Senegal river. At baseline, the percentage of urinary schistosomiasis and infection intensity in the villages studied were in line with an overall trend of a reduction in prevalence in the studied area. Indeed, 14 years ago, before regular MDA programmes were conducted, very high prevalence, between 89 and 95%, was reported in school-aged children from the Lac de Guiers and the River Doue areas, based on data collected in 2006 and 2007 [24]. After 5 or 6 years of repeated MDA programmes, prevalence dropped in several villages established in the Senegal river basin. Indeed, while in 2016, a *S. haematobium* prevalence of 88% was reported in children and adults from six villages including the village of Mbane [26], in 2017, a 66.7% overall prevalence was reported in six villages located along the Senegal river and its tributaries and ten other located on the shores of Lac de Guiers, excluding the village of Mbane [38]. In this study, the prevalence of *S. haematobium* in children from the village of Mbane using the Lac de Guiers and children living in Ndiawara and Dioundou using the river were relatively low (between 38 and 46%) compared to previous high prevalence rates [24]. We expected to find a high prevalence, since the urine examination conducted to calculate the baseline prevalence took place after the highest transmission period of *S. haematobium* (around May–July) in the Lac de Guiers area and along the Senegal river [35]. However, it appears that the decline in prevalence in the Senegal river basin began a few years ago, before our study, as indicate by previous studies [26, 38]. The low prevalence obtained in our study suggests that changes in the epidemiological situation of urinary schistosomiasis and intestinal schistosomiasis are under way in several villages in the Senegal river basin, due to the MDA programmes conducted by the Senegalese NSCP, which began in 2009 and targets school-aged children with therapeutic coverage of around 90% [25]. A recent study in sub-Saharan Africa comparing the situation before regular MDA programmes (2000–2010) and after MDA programmes (2011–2019) demonstrated that countries which undertook multiple rounds of preventive chemotherapy with high coverage over a 3-year period showed a considerable decline in the prevalence of schistosomiasis [44]. Unlike the situation in the villages bordering the Lac de Guiers and the river, our results showed that in the populations we studied, the burden of *S. haematobium* is highest in children living in villages bordering the irrigation canal. Indeed, in the villages of Guia and Khodit, the prevalence and intensity of infection remains very high, with prevalence of over 90%. This dramatic situation is similar to that which was reported before regular MDA programmes in 2007–2008 by Webster et al. [23], with a very high prevalence (95%) of *S. haematobium*. Early studies in the Senegal river basin reported that people who practice irrigated agriculture are more infected than others [45]. The very high prevalence of schistosomiasis and the intensity of infection in children living in the villages using the irrigation canals indicate that there are still significant transmission hotspots in the Senegal river basin, despite repeated MDA programmes. This means that these communities are consistently exposed to *S. haematobium* due to the proximity of the primary canal to people’s homes, as well as greater use of the canal by the population as part of their daily recreational or household activities, via the multiple points of contact with the water which exist along the canal. Several studies have established that the proximity of the households and or schools to natural water sources such as lakes, rivers and ponds is directly linked to the prevalence and intensity of the disease in sub-Saharan Africa [46, 47]. Targeting schistosomiasis at the village level, or by considering the nature and ecology of the transmission sites is an important way of orienting and adapting the strategy of intervention in order to achieve the NSCP objective, which is the long-term reduction of prevalence and morbidity of schistosomiasis.

Our findings suggest that conducting MDA programmes once every 2 years in school-aged children according to the WHO recommendation, in the villages of Mbane, Dioundou and Ndiawara, whose inhabitants use the lake and the river, is necessary [48]. However, in the villages of Guia and Khodit, whose populations frequent the irrigation canal, we suggest adapting the treatment strategy by administering a double treatment every 6 months in school-aged children and at-risk adults.

The second objective of this study was to assess the efficacy of PZQ treatment and the pattern of re-infection. Four weeks after treatment, the prevalence and intensity of *S. haematobium* drastically dropped in all villages. Very high CRs (> 96%) and ERRs (> 98%) were obtained in children from the sites near the Lac de Guiers and the river sites, where baseline prevalence and intensity were low. Even in the villages using the irrigation canal, where baseline prevalence and intensity of infection were very high, heavy intensity infections were almost completely eliminated, with an ERR of 96.9% and a satisfactory CR of 88.7%. This meets the aim of reducing morbidity and thus, confirms the efficacy of a single dose of 40 mg/kg PZQ for the treatment of urinary schistosomiasis. A previous study reported a CR > 80% 6 weeks after treatment in four villages in the Podor area, with marked declines in levels of infection [22]. Webster et al*.* reported similar high ERR rates (97.8%) in children from the village of Guia 6 weeks after the administration of two rounds of 40 mg/kg at 3-week intervals, while a low CR (47.1%) was obtained [23]. Our satisfactory CR may be associated with the fact that even children for whom a single miracidia was detected in the urine sample after treatment were considered to be uncured. Indeed, if a single egg in the parasitological sample is considered to be a failure of cure, without egg hatching for confirmation, this can lead to low CR but high ERR [49, 50]. However, several non-exclusive factors including, for instance, the transmission dynamic of *S. haematobium*, the baseline prevalence and infection intensity, the age of the targeted population, the PZQ dose, the timing of PZQ efficacy diagnosis, and the rapidity of re-infection could explain the differences in the CR and EER observed between studies within and between countries [12, 23, 49, 51, 52]. In the case of our study, as there was insufficient time for re-infection between treatment and assessment time to result in egg production [17, 23], the 13/16 failed cases observed after treatment in the villages near the irrigation canal were probably the result of the maturation of pre-existing juvenile parasites, since PZQ has no effect on them [53]. Alternatively, this result could also reflect a higher resistance among some parasite genotypes to the treatment. In general, it is difficult to obtain 100% CR and ERR following treatment with PZQ, even in areas with low prevalence and infection intensity. Some parasites that are not completely killed can continue excreting viable eggs even after three doses of PZQ [40]. To explain this, parameters such as infection intensity, low drug absorption, high levels of catabolism, rapid re-infection, and the lack of acquired immunity have often been advocated [23, 54]. It is thus very difficult to tease apart the possible resistance to treatment from treatment failure in the field. Drug-induced resistance can only be confirmed by linking changes in resistance allele frequencies to phenotypic indicators of drug tolerance or reduced susceptibility [55]. It is very important to monitor PZQ resistance given the drug pressure in endemic areas in sub-Saharan Africa, in particular in the Senegal river basin [10, 49]. In this regard, hybridisation between Schistosoma species have been repeatedly documented in this geographical area [26, 56–58] and are thought to potentially promote the emergence of hybrid-resistant strains of schistosomes [59–62]. Thus, the genomic comparison of parasite communities before and after several rounds of treatment using the next generation sequencing approach is indispensable and will enable the detection of possible resistance markers of Schistosome to PZQ by scanning whole genomes [55, 63].

One major obstacle in the fight against schistosomiasis in SSA is the fact that a patient who has just been cured of infection can be almost instantaneously reinfected after contact with infested water. In our study, re-infection occurred 7 months after treatment, and reflected the variation in the baseline prevalence in the different epidemiological systems, with the highest rate (42.4%) in the villages near the irrigation canal and the lowest rates in those close to the Lac de Guiers (18.4%) and the river (14.8%). Nevertheless, the infection levels were still markedly reduced compared to pre-treatment in all villages. Similar results were reported by Shaw et al*.* in the middle valley, in the villages of Diatar and Guia, where MDA was administered, while in Donaye and Niandane where MDA was not administered, prevalence levels had returned to pre-treatment levels after 6 months [30]. Webster et al. [23] also reported, in a cohort of children in 2009 and 2010, that 6 months after treatment, the prevalence after re-infection approached the baseline in the village of Guia whose population frequented the irrigation canal. Schistosomiasis transmission and re-infection are known to feature focal differentiation in endemic areas, as transmission is regulated by the distribution and dynamics of the snail intermediate hosts in freshwater, which depends on the environmental and ecological conditions. Therefore, the transmission of schistosomiasis could vary from one area to another and even within the same endemic villages from 1 year to the next [52, 64]. In the river and the Lac de Guiers, *S. haematobium* transmission is perennial, as water is present all year long, while the canals may dry depending on agricultural activities in the farms [35]. The highest rate of re-infection observed in children from Guia and Khodit who used the canal could be due to a high exposure of the population due to their proximity and frequency of contact with the canal water as well as its accessibility. However, these locations also present all the environmental conditions that are favourable for the snail host populations and/or for the parasites to be transmitted, thus constituting important transmission hotspots. In our study, re-infection was checked at the end of the high transmission season in March–April, 7 months after treatment. At this time point, any *S. haematobium* parasites that had reinfected the children should be adults and should have started to excrete eggs in the urine. However, by comparing our results to those previously reported in the Senegal river basin, it would appear that there is a new pattern of re-infection, which is not rapid, with low to moderate rates, contrary to the previous year, where re-infection was rapid and could sometimes reach baseline prevalence [22, 23]. Overall, our results show that regular annual MDA programmes have had an impact on reducing the prevalence and intensity of infection, and have limited the re-infection rates of *S. haematobium* in the Senegal river basin. So while waiting for the development and roll-out of a future anti-schistosome vaccine, which will contribute greatly to the ultimate elimination of the disease [65], a multisectoral approach will be required. This will include epidemiological surveillance, effective risk mapping, correctly timing control measures on the snail intermediate hosts, adapting the treatment regimen at the village level, improving access to clean water, sanitation and hygiene, and public health education to induce behavioural change and prevent infection and re-infection [36, 66, 67].

## Conclusion

Ours study confirms the efficacy of the standard dose of 40 mg/kg of praziquantel in the treatment of *S. haematobium* infection in the several villages associated with different transmission site profiles along the Senegal river basin. The prevalence and intensity of infection is generally lower than 10 years previously, before the regular annual mass administration of PZQ by the Senegalese NSCP in school-aged children in the villages using the river and the Lac de Guiers, while the villages frequenting the irrigation canals remain hotspots of urogenital schistosomiasis. Thus, a large investigation of prevalence by NSCP through the school system could be a good means of adapting the WHO recommendations to the local situation. Due to the high level of re-infection observed 7 month after the first treatment in the villages near the irrigation canals, we recommend two MDA programmes at a 6-month interval in these villages, while maintaining the annual single treatment in the other villages in accordance with the WHO guideline on control and elimination of schistosomiasis [68] (Additional file 3).

## Supplementary Information

**Additional file 1: Figure S1.** Baseline prevalence (a) and infection intensity (b) according to the type of water access.

**Additional file 2: Table S1.** Tables of anova tests

**Additional file 3.** Datasheets.

## Data Availability

The datasets generated during and/or analyzed during the current study are available in the Additional file 3.

## Abbreviations

PZQ: Praziquantel
MDA: Mass drug administration
SSA: Sub-Saharan Africa
WHO: World Health Organization
CR: Cure rate
ERR: Egg reduction rate
AMEC: Arithmetic Mean Eggs Count
NSCP: National schistosomiasis control program
glmm: Generalized linear mixed models

## References

[1] Vos T, Flaxman AD, Naghavi M, Lozano R, Michaud C, Ezzati M (2012). Years lived with disability (YLDs) for 1160 sequelae of 289 diseases and injuries 1990–2010: a systematic analysis for the Global Burden of Disease Study 2010. Lancet.

[2] GBD DALYs and HALE Collaborators (2016). Global, regional, and national disability-adjusted life-years (DALYs) for 333 diseases and injuries and healthy life expectancy (HALE) for 195 countries and territories, 1990–2016: a systematic analysis for the Global Burden of Disease Study 2016. Lancet.

[3] van der Werf MJ, de Vlas SJ, Brooker S, Looman CWN, Nagelkerke NJD, Habbema JDF (2003). Quantification of clinical morbidity associated with schistosome infection in sub-Saharan Africa. Acta Trop.

[4] Gryseels B, Polman K, Clerinx J, Kestens L (2006). Human schistosomiasis. Lancet.

[5] Grimes JET, Croll D, Harrison WE, Utzinger J, Freeman MC, Templeton MR (2014). The relationship between water, sanitation and schistosomiasis: a systematic review and meta-analysis. PLoS Negl Trop Dis.

[6] Fenwick A, Savioli L, Engels D, Robert Bergquist N, Todd MH (2003). Drugs for the control of parasitic diseases: current status and development in schistosomiasis. Trends Parasitol.

[7] Hotez PJ, Kamath A (2009). Neglected tropical diseases in sub-saharan Africa: review of their prevalence, distribution, and disease burden. PLoS Negl Trop Dis.

[8] Utzinger J, Raso G, Brooker S, De Savigny D, Tanner M, Ornbjerg N (2009). Schistosomiasis and neglected tropical diseases: towards integrated and sustainable control and a word of caution. Parasitology.

[9] WHO (2020). Schistosomiasis and soil-transmitted helminthiases: numbers of people treated in 2019. Wkly Epidemiol Rec.

[10] Fallon PG, Doenhoff MJ (1994). Drug-resistant schistosomiasis: resistance to praziquantel and oxamniquine induced in *Schistosoma mansoni* in mice is drug specific. Am J Trop Med Hyg.

[11] Ahmed AM, El Tash LA, Mohamed EY, Adam I (2012). High levels of *Schistosoma mansoni* infections among schoolchildren in central Sudan one year after treatment with praziquantel. J Helminthol.

[12] Mutapi F, Rujeni N, Bourke C, Mitchell K, Appleby L, Nausch N (2011). Schistosoma haematobium treatment in 1–5 year old children: safety and efficacy of the antihelminthic drug praziquantel. PLoS Negl Trop Dis.

[13] Danso-Appiah A, De Vlas SJ (2002). Interpreting low praziquantel cure rates of *Schistosoma mansoni* infections in Senegal. Trends Parasitol.

[14] Talla I, Kongs A, Verlé P, Belot J, Sarr S, Coll AM (1990). Outbreak of intestinal schistosomiasis in the Senegal River Basin. Ann Soc Belg Med Trop.

[15] Chaine JP, Malek EA (1983). Urinary schistosomiasis in the Sahelian region of the Senegal River Basin. Trop Geogr Med.

[16] Vercruysse J, Southgate VR, Rollinson D (1985). The epidemiology of human and animal schistosomiasis in the Senegal River Basin. Acta Trop.

[17] Picquet M, Ernould JC, Vercruysse J, Southgate VR, Mbaye A, Sambou B (1996). The epidemiology of human schistosomiasis in the Senegal river basin. Trans R Soc.

[18] Diaw OT, Vassiliades G, Seye M, Sarr Y (1990). Prolifération de mollusques et incidence sur les trématodoses dans la région du delta et du lac de Guiers après la construction du barrage de Diama sur le fleuve Sénégal. Revue Élev Méd vét Pays trop.

[19] Southgate VR (1997). Schistosomiasis in the Senegal River Basin: before and after the construction of the dams at Diama, Senegal and Manantali, Mali and future prospects. J Helminthol.

[20] De Clercq D, Vercruysse J, Picquet M, Shaw DJ, Diop M, Ly A (1999). The epidemiology of a recent focus of mixed *Schistosoma haematobium* and *Schistosoma*
*mansoni* infections around the “Lac de Guiers” in the Senegal River Basin, Senegal. Trop Med Int Health.

[21] Southgate VR, Tchuem Tchuenté L-A, Sène M, De Clercq D, Théron A, Jourdane J (2001). Studies on the biology of schistosomiasis with emphasis on the Senegal river basin. Mem Inst Oswaldo Cruz.

[22] Shaw DJ, Vercruysse J, Picquet M, Sambou B, Ly A (1999). The effect of different treatment regimens on the epidemiology of seasonally transmitted *Schistosoma*
*haematobium* infections in four villages in the Senegal River Basin, Senegal. Trans R Soc Trop Med Hyg.

[23] Webster BL, Diaw OT, Seye MM, Faye DS, Stothard JR, Sousa-Figueiredo JC (2013). Praziquantel treatment of school children from single and mixed infection foci of intestinal and urogenital schistosomiasis along the Senegal River Basin: monitoring treatment success and re-infection patterns. Acta Trop.

[24] Boon NAM, Broeck FVD, Faye D, Volckaert FAM, Mboup S, Polman K (2018). Barcoding hybrids: heterogeneous distribution of *Schistosoma haematobium* × *Schistosoma bovis* hybrids across the Senegal River Basin. Parasitology.

[25] Abdellahi M, Ndir O, Niang S (2016). Évaluation de la prévalence des bilharzioses auprès des enfants de 5 à 14 ans après plusieurs années de traitement de masse dans le bassin du fleuve Sénégal. Sante Publique.

[26] Leger E, Borlase A, Fall CB, Diouf ND, Diop SD, Yasenev L (2020). Prevalence and distribution of schistosomiasis in human, livestock, and snail populations in northern Senegal: a One Health epidemiological study of a multi-host system. Lancet Planet Health.

[27] De Clercq D, Vercruysse J, Kongs A, Verlé P, Dompnier JP, Faye PC (2002). Efficacy of artesunate and praziquantel in *Schistosoma haematobium* infected schoolchildren. Acta Trop.

[28] Guisse F, Polman K, Stelma FF, Mbaye A, Talla I, Niang M (1997). Therapeutic evaluation of two different dose regimens of praziquantel in a recent *Schistosoma*
*mansoni* focus in Northern Senegal. Am J Trop Med Hyg.

[29] Picquet M, Vercruysse J, Shaw DJ, Diop M, Ly A (1998). Efficacy of praziquantel against *Schistosoma mansoni* in northern Senegal. Trans R Soc Trop Med Hyg.

[30] Stelma FF, Talla I, Sow S, Kongs A, Niang M, Polman K (1995). Efficacy and side effects of praziquantel in an epidemic focus of *Schistosoma*
*mansoni*. Am J Trop Med Hyg.

[31] Alonso D, Muñoz J, Gascón J, Valls ME, Corachan M (2006). Failure of standard treatment with praziquantel in two returned travelers with *Schistosoma haematobium* infection. Am J Trop Med Hyg.

[32] Doenhoff MJ, Kusel JR, Coles GC, Cioli D (2002). Resistance of *Schistosoma mansoni* to praziquantel: is there a problem?. Trans R Soc Trop Med Hyg.

[33] da Silva IM, Thiengo R, Conceição MJ, Rey L, Lenzi HL, Pereira Filho E (2005). Therapeutic failure of praziquantel in the treatment of *Schistosoma*
*haematobium* infection in Brazilians returning from Africa. Mem Inst Oswaldo Cruz.

[34] Diaw OT. Population dynamics of schistosome intermediate host snails in a village in the delta of the Senegal River basin. In: Proceedings of ‘Workshop on Medical Malacology in Africa.’ Harare: L.N.E.R/lSRA/Service de Parasitologie; 1997. p. 173–84.

[35] Sturrock RF, Diaw OT, Talla I, Niang M, Piau JP, Capron A (2001). Seasonality in the transmission of schistosomiasis and in populations of its snail intermediate hosts in and around a sugar irrigation scheme at Richard Toll, Senegal. Parasitology.

[36] Jones IJ, Sokolow SH, Chamberlin AJ, Lund AJ, Jouanard N, Bandagny L (2021). Schistosome infection in Senegal is associated with different spatial extents of risk and ecological drivers for *Schistosoma*
*haematobium* and *S. mansoni*. PLOS Negl Trop Dis.

[37] Sow S, de Vlas SJ, Stelma F, Vereecken K, Gryseels B, Polman K (2011). The contribution of water contact behavior to the high *Schistosoma mansoni* infection rates observed in the Senegal River Basin. BMC Infect Dis.

[38] Lund AJ, Sokolow SH, Jones IJ, Wood CL, Ali S, Chamberlin A (2021). Exposure, hazard, and vulnerability all contribute to *Schistosoma haematobium* re-infection in northern Senegal. PLoS Negl Trop Dis.

[39] Plouvier S, Leroy JC, Colette J (1975). A propos d’une technique simple de filtration des urines dans le diagnostic de la bilharziose urinaire en enquête de masse. Med Trop.

[40] Kabuyaya M, Chimbari MJ, Manyangadze T, Mukaratirwa S (2017). Efficacy of praziquantel on *Schistosoma haematobium* and re-infection rates among school-going children in the Ndumo area of uMkhanyakude district, KwaZulu-Natal, South Africa. Infect Dis Poverty.

[41] Tchuem Tchuenté L-A, Momo SC, Stothard JR, Rollinson D (2013). Efficacy of praziquantel and reinfection patterns in single and mixed infection foci for intestinal and urogenital schistosomiasis in Cameroon. Acta Trop.

[42] Montresor A, Crompton DWT, Hall A, Bundy DAP, Savioli L, Unit WHOD of C of TDS and IP. Guidelines for the evaluation of soil-transmitted helminthiasis and schistosomiasis at community level: a guide for managers of control programmes. 1998.

[43] Bolker B, Skaug H, Magnusson A, Nielsen A. Getting started with the glmmADMB package. 2012.

[44] Kokaliaris C, Garba A, Matuska M, Bronzan RN, Colley DG, Dorkenoo AM (2021). Effect of preventive chemotherapy with praziquantel on schistosomiasis among school-aged children in sub-Saharan Africa: a spatiotemporal modelling study. Lancet Infect Dis.

[45] de Clercq D, Vercruysse J, Sène M, Seck I, Sall CS, Ly A (2000). The effects of irrigated agriculture on the transmission of urinary schistosomiasis in the Middle and Upper Valleys of the Senegal River basin. Ann Trop Med Parasitol.

[46] Adenowo AF, Oyinloye BE, Ogunyinka BI, Kappo AP (2015). Impact of human schistosomiasis in sub-Saharan Africa. Braz J Infect Dis.

[47] Kapito-Tembo AP, Mwapasa V, Meshnick SR, Samanyika Y, Banda D, Bowie C (2009). Prevalence distribution and risk factors for *Schistosoma*
*hematobium* infection among school children in Blantyre, Malawi. PLoS Negl Trop Dis.

[48] OMS. Schistosomiase: Rapport de situation 2001–2012: plan stratégique 2012–2020. Rapport de situation 2001–2012: plan stratégique 2012–2020. Genève: Organisation mondiale de la santé; 2013.

[49] Fukushige M, Chase-Topping M, Woolhouse MEJ, Mutapi F (2021). Efficacy of praziquantel has been maintained over four decades (from 1977 to 2018): a systematic review and meta-analysis of factors influence its efficacy. PLoS Negl Trop Dis.

[50] Midzi N, Sangweme D, Zinyowera S, Mapingure MP, Brouwer KC, Kumar N (2008). Efficacy and side effects of praziquantel treatment against *Schistosoma*
*haematobium* infection among primary school children in Zimbabwe. Trans R Soc Trop Med Hyg.

[51] N’Goran EK, Utzinger J, N’Guessan AN, Müller I, Zamblé K, Lohourignon KL (2001). Reinfection with *Schistosoma haematobium* following school-based chemotherapy with praziquantel in four highly endemic villages in Côte d’Ivoire. Trop Med Int Health.

[52] Senghor B, Diaw OT, Doucoure S, Sylla SN, Seye M, Talla I (2015). Efficacy of praziquantel against urinary schistosomiasis and reinfection in Senegalese school children where there is a single well-defined transmission period. Parasit Vectors.

[53] Utzinger J, N’Goran EK, N’Dri A, Lengeler C, Xiao S, Tanner M (2000). Oral artemether for prevention of *Schistosoma mansoni* infection: randomised controlled trial. Lancet.

[54] Gryseels B, Stelma FF, Talla I, van Dam GJ, Polman K, Sow S (1994). Epidemiology, immunology and chemotherapy of Schistosoma mansoni infections in a recently exposed community in Senegal. Trop Geogr Med.

[55] Crellen T, Walker M, Lamberton PHL, Kabatereine NB, Tukahebwa EM, Cotton JA (2016). Reduced efficacy of praziquantel against *Schistosoma mansoni* Is associated with multiple rounds of mass drug administration. Clin Infect Dis.

[56] Huyse T, Webster BL, Geldof S, Stothard JR, Diaw OT, Polman K (2009). Bidirectional introgressive hybridization between a cattle and human schistosome species. PLoS Pathog.

[57] Sene M, Marchand B, Rollinson D, Webster B (2018). Urogenital schistosomiasis and hybridization between *Schistosoma haematobium* and *Schistosoma bovis* in adults living in Richard-Toll, Senegal. Parasitology.

[58] Webster BL, Diaw OT, Seye MM, Webster JP, Rollinson D (2013). Introgressive hybridization of *Schistosoma haematobium* group species in Senegal: species barrier break down between ruminant and human schistosomes. PLoS Negl Trop Dis.

[59] Arnold ML (2004). Natural hybridization and the evolution of domesticated, pest and disease organisms. Mol Ecol.

[60] Borlase A, Rudge JW, Léger E, Diouf ND, Fall CB, Diop SD (2021). Spillover, hybridization, and persistence in schistosome transmission dynamics at the human–animal interface. PNAS.

[61] Detwiler JT, Criscione CD (2010). An infectious topic in reticulate evolution: introgression and hybridization in animal parasites. Genes (Basel).

[62] Leger E, Webster JP (2017). Hybridizations within the Genus *Schistosoma*: implications for evolution, epidemiology and control. Parasitology.

[63] Lamberton PHL, Hogan SC, Kabatereine NB, Fenwick A, Webster JP (2010). In vitro praziquantel test capable of detecting reduced in vivo efficacy in *Schistosoma mansoni* human infections. Am J Trop Med Hyg.

[64] Clennon JA, King CH, Muchiri EM, Kariuki HC, Ouma JH, Mungai P (2004). Spatial patterns of urinary schistosomiasis infection in a highly endemic area of coastal Kenya. Am J Trop Med Hyg.

[65] Aula OP, McManus DP, Jones MK, Gordon CA (2021). Schistosomiasis with a focus on Africa. Trop Med Infect Dis.

[66] Campbell SJ, Savage GB, Gray DJ, Atkinson J-AM, Soares Magalhães RJ, Nery SV (2014). Water, sanitation, and hygiene (WASH): a critical component for sustainable soil-transmitted helminth and schistosomiasis control. PLoS Negl Trop Dis.

[67] Cioli D, Pica-Mattoccia L, Basso A, Guidi A (2014). Schistosomiasis control: praziquantel forever?. Mol Biochem Parasitol.

[68] WHO (2022). WHO guideline on control and elimination of human schistosomiasis.

