# Supplementary figures and images for: Urogenital schistosomiasis in three different water access in the Senegal river basin: prevalence and monitoring praziquantel efficacy and re-infection levels
Source: BMC Infect Dis. 2022 Dec 29;22:968. doi: 10.1186/s12879-022-07813-5 (PMC9801593; doi:10.1186/s12879-022-07813-5)

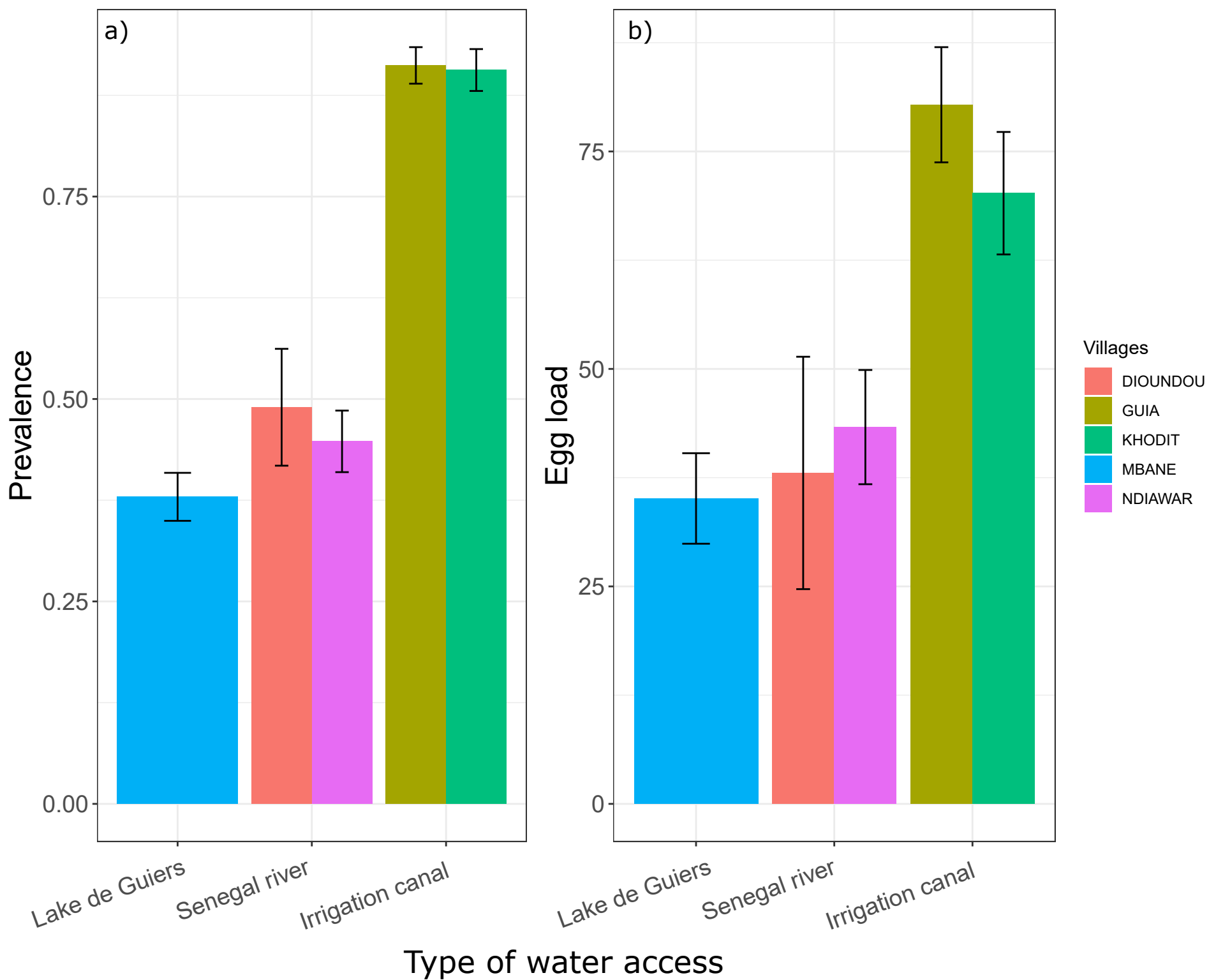

Supplement: Supplementary file 1 — Additional file 1: Figure S1. Baseline prevalence (a) and infection intensity (b) according to the type of water access. [file 12879_2022_7813_MOESM1_ESM.pdf]
