# Supplementary material for: Urogenital schistosomiasis in three different water access in the Senegal river basin: prevalence and monitoring praziquantel efficacy and re-infection levels
Source: BMC Infect Dis. 2022 Dec 29;22:968. doi: 10.1186/s12879-022-07813-5 (PMC9801593; doi:10.1186/s12879-022-07813-5)
Supplement: Supplementary file 2 — Additional file 2: Table S1. Tables of anova tests [file 12879_2022_7813_MOESM2_ESM.docx]

Table S1 : Tables of anova tests

| Egg load | variables | F statistic | d.f. | p-values |
| --- | --- | --- | --- | --- |
|  | Water access type | 18,690 | 2 | <0.001 |
|  | Age | 0,003 | 1 | 0,960 |
|  | Sex | 0,025 | 1 | 0,874 |
| Prevalence | variables | X² statistic | d.f. | p-values |
|  | Water access type | 172,4718 | 2 | <0.001 |
|  | Age | 0,9714 | 1 | 0,324 |
|  | Sex | 0,9714 | 1 | 0,424 |
